# The Effect of Hypoxia-Induced Exosomes on Anti-Tumor Immunity and Its Implication for Immunotherapy

**DOI:** 10.3389/fimmu.2022.915985

**Published:** 2022-06-22

**Authors:** Wenwen Guo, Tianyun Qiao, Bingwei Dong, Tian Li, Qiang Liu, Xiaofeng Xu

**Affiliations:** ^1^ Clinical Research Center, Xianyang Central Hospital, Xianyang, China; ^2^ Department of Thoracic Surgery, Tangdu Hospital, Fourth Military Medical University, Xi’an, China; ^3^ School of Basic Medicine, Fourth Military Medical University, Xi’an, China

**Keywords:** hypoxia, exosomes, immunotherapy, tumor microenvironment, anti-tumor immunity

## Abstract

Hypoxia is a critical feature of solid tumors and is considered to be a key factor in promoting tumorigenesis and progression. Beyond inducing metabolic reprogramming of tumor cells to adapt to the hypoxia tumor microenvironment (TME), hypoxia can also promote tumor growth by affecting the secretion of exosomes. Exosomes are nano-sized (30-150 nm in diameter) extracellular vesicles that can carry numerous substances including lipids, proteins, nucleic acids, and metabolites. Notably, hypoxia-induced exosomes alterations not only exist in tumor cells, but also in various TME cells including stromal cells and immune cells. Besides promoting tumor invasion, angiogenesis, and drug resistance, the secretion of these altered exosomes has recently been found to negatively regulate anti-tumor immune responses. In this review, we focus on the hypoxia-induced changes in exosome secretion and found it can contributes to immune evasion and cancer progression by recruiting protumor immune cells into TME, as well as inhibiting antitumor immune cells. Next, we also describe the recent advances of exosomes in immunotherapy and future direction. In conclusion, ongoing discoveries in this field have brought new insights into hypoxia exosome-led immunosuppression, enabling the development of exosome-based therapeutics and elucidating their potential in immunotherapy.

## Introduction

The tumor microenvironment (TME) is a complex and highly heterogeneous environment, which is composed of blood vessels, immune cells, fibroblasts, extracellular matrix, signaling molecules (i.e., chemokines, cytokines, growth factors, etc.), and metabolic wastes (e.g., lactic acid) (1). Hypoxia (low oxygen concentration) is a major feature of the TME in most solid tumors and has been reported to be associated with tumor progression, therapy resistance, and poor clinical prognosis (2). Hypoxia is caused by the increased oxygen demand of rapid tumor tissue proliferation and insufficient oxygen supply due to tumor vascular defects. In general, tumors can improve oxygen supply by activating hypoxia-inducible factor (HIF) to promote tumor neo-angiogenesis (3). Notably, hypoxia not only regulates tumor angiogenesis and metabolic reprogramming but also mediates tumor immune escape, invasion, and metastasis, as well as therapeutic drug resistance (4). Therefore, in the past decades, hypoxia TMEs have received extensive research attention and are regarded as an important target for tumor therapy. Drugs that improve tumor hypoxia, such as bevacizumab and topotecan, have been widely used in the clinical treatment of various tumors and significantly improve the prognosis of patients (5). Noteworthy, a growing number of new findings indicate that hypoxia can also affect tumor growth, invasion, and metastasis, as well as anti-tumor immunity by regulating the secretion of exosomes in the TME (6). Therefore, targeting hypoxia-induced exosomes may be the next key breakthrough in ameliorating the adverse effects of tumor hypoxia, as well as promoting the effect of tumor immunotherapy.

Exosomes are nanoscale bilayer vesicles released by various cell types (tumor cells, stromal cells, immune cells, etc.) upon fusion of multivesicular bodies with the plasma membrane (7). They carry various genetic information from parental cells and are deeply involved in the exchange of information between cells (8, 9). Therefore, the size and cargo of an exosome are directly determined by its cell of origin (10, 11). In addition, the size and cargo variables are also greatly affected by TME, such as hypoxia and acidic microenvironment (12). However, the exact mechanism of the association between exosomes and hypoxia during tumor progression needs further elucidation. Studies have shown that typical exosomes have a diameter of 30-150 nm and are usually cup-shaped (13). They all contain multiple types of proteins (such as Rab GTPases, annexin, heat shock proteins HSP60 and HSP905-7) and lipids (e.g., ceramides, cholesterol, and glycerophospholipids), nucleic acids (i.e., mRNAs, microRNAs, circRNAs, and long non-coding RNAs), and metabolites (14, 15). They are present in almost all body fluids, including blood, sweat, tears, urine, ascites, to cerebrospinal fluid. Exosomes were originally thought to be the “dumpster” of cells (16). This view remained unchanged until 1996 when Raposo et al. found that exosomes can affect the function of immune cells (17). However, subsequent studies on exosome functions have revealed their critical roles in intercellular communication, antigen presentation, cell differentiation, anti-tumor immune response, tumor cell migration, and invasion (15). Therefore, the exact mechanism of the association between exosomes and hypoxia during tumor progression needs further elucidation.

## Regulation of Hypoxia on Exosomes

As mentioned above, the hypoxia TME can regulate processes such as exosome formation, loading, and release of cargo, which in turn affects intercellular communication at local and distant sites. Multiple studies have shown that tumor cells regulate the secretion of exosomes, as well as the size and distribution of exosomes through HIF-1 under hypoxia conditions (18). For instance, Li et al. found a marked increase in the number of miRNAs in exosomes secreted by oral squamous cell carcinoma (OSCC) cells under hypoxia conditions. Further mechanistic studies found that the up-regulation of miRNAs in exosomes, especially miRNA-21, was mediated by HIF-1α and HIF-2α, while closely related to tumor stage and lymph node metastasis in patients with OSCC (19). Similarly, Wang et al. exposed three human breast cancer cell lines to a hypoxia environment (1% O_2_, 24 h) and examined the changes in exosome secretion. Results showed that the number of exosomes was significantly increased under the regulation of the HIF-1α-dependent small GTPase Rab22A (20). In addition, Huang et al. found that HIF-1α can regulate the expression of miRNA-210 in a variety of tumors through hypoxia response elements (21). Noteworthy, other studies have also reported that miRNA-210 is also up-regulated in multiple tumors and is therefore considered to be the most extensive miRNA in hypoxia-induced exosomes (22, 23). In ovarian cancer, HIF induces the release of exosomes with elevated levels of multiple miRNAs, such as miR-21-3p, miR-125b-5p, and miR-181d-5p. Further *in vivo* studies found that these hypoxia-induced exosomes can promote tumor proliferation and migration by inducing M2 polarization of macrophages (24). Notably, hypoxia-induced increased exosome secretion is not a phenomenon unique to tumor cells, as Zhang et al. observed HIF-1-mediated increased exosome production and secretion in renal proximal tubule cells under hypoxia (25). Recent studies have also shown that mesenchymal stem cells (MSCs) can also promote the secretion of miRNA-126 in exosomes through HIF-1α in hypoxia (26).

Increasing evidences have shown that increased/altered exosomal protein content also responsible for tumorigenesis, invasion, and drug resistance. Notably, several studies further reported that exosomes released from hypoxia TME are more likely to cause tumor invasion and angiogenesis. Kore et al. qualitatively and quantitatively analyzed the protein content of exosomes secreted by hypoxia-treated glioblastoma cells. The results showed significantly elevated proteins levels such as thrombospondin-1 (TSP1), vascular endothelial growth factor (VEGF), and protein-lysine 6-oxidase (LOX), which have been well documented to be associated with tumor progression, angiogenesis, and treatment resistance (27). Moreover, Huang et al. found that exosomes secreted by colorectal cancer (CRC) cell under hypoxia conditions can promote the migration and invasion of normoxic CRC cell. Further quantitative analysis found that HIF1α-dependent Wnt4 was significantly elevated in these hypoxia-induced exosomes. Elevated Wnt4 in exosomes is thought to enhance the metastatic ability of normal CRC cell, which may provide a new target for CRC therapy (28). Besides proteins and nucleic acids, lipids and metabolites are also important components of exosomes, although much less information is available on their composition and effects. For instance, Schlaepfer et al. observed markedly elevated palmitic, stearic, and linoleic acids in exosomes secreted by hypoxia-treated prostate cancer cell (29). All these studies suggest that hypoxia can affect the secretion and release of various exosome contents.

HIF has been identified to induce exosome secretion by increasing the expression and activation of cell surface receptors such as glucose transporter and transferrin receptor. Specifically, Luo et al. revealed that HIF-1 promotes aerobic glycolysis of tumor cells by up-regulating the expression of M2-type pyruvate kinase (PKM2) mRNA in the hypoxia TME (30). Furthermore, Wei et al. found that PKM2 regulates exosome release mainly by phosphorylating Ser95 of synaptosomeassociated protein 23 (SNAP-23), which is a major component of the synaptosome/SNARE complex. These finding suggests that PKM2 is not just a key enzyme in the process of aerobic glycolysis but promotes the release of exosomes under hypoxia conditions. Thus, it is not surprising that shikonin was found to reduce exosomes release by inhibiting glycolysis, whereas activation of glycolysis by tumor necrosis factor-alpha (TNF-α) increased the secretion of exosomes (31).

**Figure 1:**
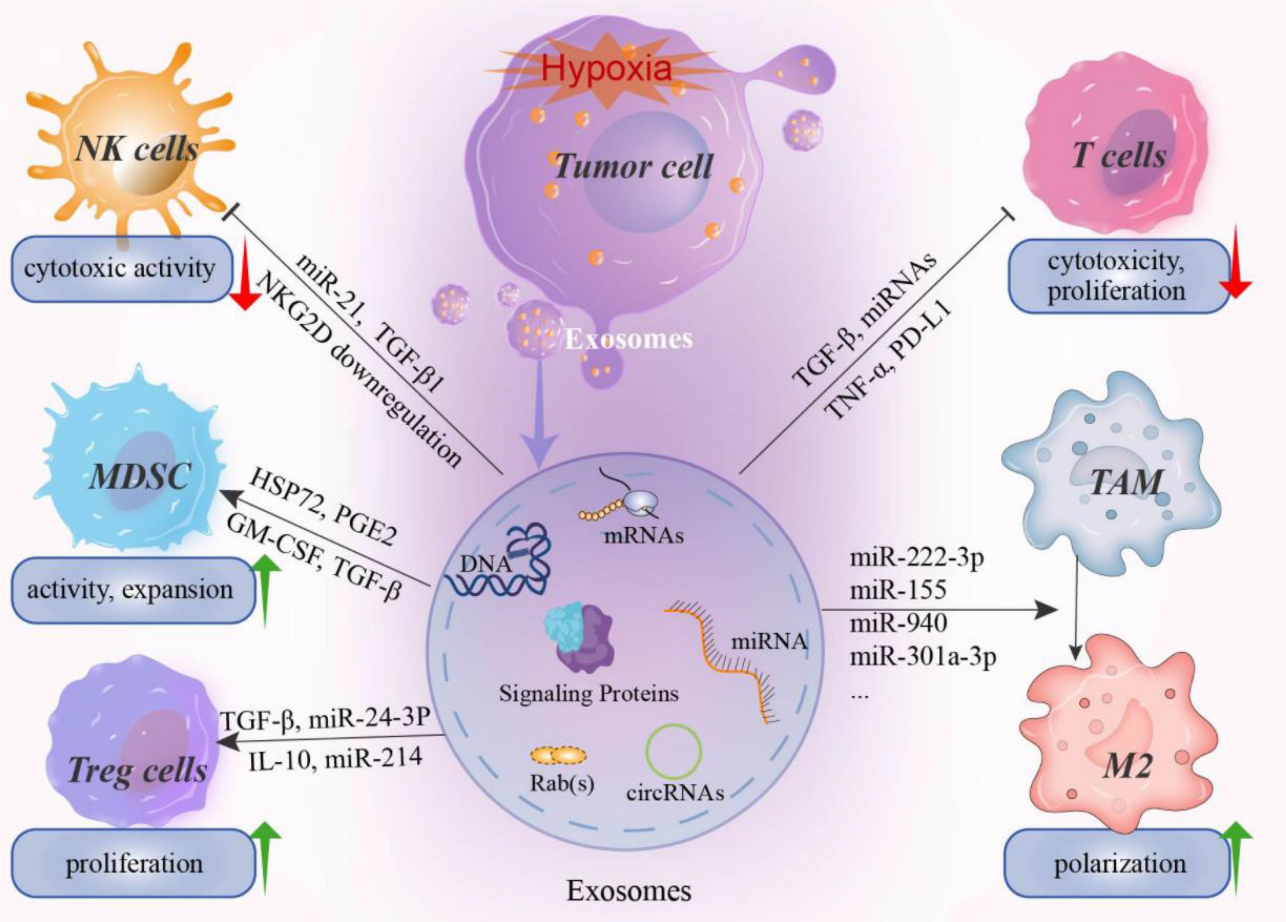
The effect of hypoxia induced exosomes on immune system. Hypoxia in the tumor microenvironment can induce tumor cells to secrete a large number of exosomes, including miRNAs, mRNAs, signaling proteins, nucleotides, and immunomodulatory factors. These hypoxia-induced exosomes can mediate the immune evasion of tumor cells by affecting the activity of immune killer cells and promoting the proliferation and activation of immunosuppressive cells. For example, miR-21 and miR-29a in hypoxia-induced exosomes can inhibit the cytotoxicity of NK cell by downregulating the activating receptor NKG2D. In addition, the proliferation and activation of cytotoxic T cells were also inhibited by exosomes. In contrast, for MDSCs, Treg cells, and TAM, hypoxia-induced exosomes can promote the expansion and transformation of these cells. For example, various miRNAs have been reported to promote the M2 polarization of TAMs and thus promote the formation of an immunosuppressive microenvironment. NK cell, natural killer cell; MDSCs, myeloid-derived suppressor cells; Treg, regulatory T cell; TAM, Tumor-associated macrophages.

In addition to HIF, other signaling molecules and pathways, such as Rab-GTPase, NF-κB, oxidative stress, and PI3K/Akt/mTOR, are also involved in biogenesis and releasing of exosomes under hypoxia (32). For instance, signal transducer and activator of transcription 3 (STAT3) promotes exosome release by down-regulating Rab7 and up-regulating Rab27a in ovarian cancer cells under hypoxia conditions (33). In addition, RAb5 has also been reported to regulate exosome release by regulating the transport of vesicles from the cell membrane to early endosomes and fusion with homotypic early endosomes (34). Moreover, the increase of reactive oxygen species under hypoxia can induce oxidative stress, which in turn promotes the release of exosomes. For example, Jurkat T cells secrete about 15 times more exosomes under oxidative stress conditions, while Raji cells secreted about 32 times more exosomes (35). Collectively, these findings confirm that the hypoxia TME can stimulate tumor cells to secrete more exosomes and thereby affect exosome cargo loading. However, the specific molecular mechanism by which hypoxia regulates exosome release is worth further exploration (**Figure 1**).

## Regulation of the Immune System by Hypoxia-Induced Exosomes

It is well known that exosomes are responsible for intercellular communication that can influence the development, maturation, and anti-tumor activity of immune cells by regulating their molecular signaling (36, 37). On the one hand, under normoxia, exosome secretions can directly activate immune effector cells *in vivo* and induce stronger immune responses. For example, HSP70 on the surface of exosomes can stimulate the activation of natural killer (NK) cells and macrophages, as well as induce stronger T cell responses (38). The exosomes released by mature dendritic cells (DCs) contain elevated levels of MHC I, MHC II, and co-stimulatory molecules, which showed a stronger effect on antigen presentation and immune stimulation (39). In addition, DC-derived exosomes can also express IL-15Rα and natural killer group 2D (NKG2D) ligands, which can promote the proliferation and activation of CD8^+^ T and NK cells respectively (40, 41). In view of the DC-derived exosomal immunogenicity and immune activation, numerous exosome-based anti-tumor vaccines have entered phase I and phase II clinical trials. On the other hand, tumor-derived exosomes, especially hypoxia-induced exosomes, are rich in a variety of immunomodulatory proteins and chemokines, including CSF-1, CCL2, FTH, FTL, IL-10, and TGF. These hypoxia-induced exosomes promoted the generation and infiltration of immunosuppressive T regulatory (Treg) cells, promoted the polarization of M2-macrophages, and inhibited the proliferation of T cells, collectively promote the immune evasion of tumors (42). In addition, studies have shown that programmed death protein ligand 1 (PD-L1) contained in tumor cell-derived exosomes can bind to programmed death protein 1 (PD-1) receptors on T cells to inhibit T cell activation, thereby promoting immune escape of tumors (43).

In general, recent studies on hypoxia-induced exosomes have found that inhibition of immune cells in the TME may be the main reason for the failure of anti-tumor immunity. Therefore, clarifying the regulatory role of exosomes on various immune cells in the hypoxia TME is particularly important for the development of more effective and precise immunotherapy methods (**Table 1**).

**Table 1:** Hypoxia-induced exosomes involved in anti-tumor immunity.

**Regulatory factors**	**Cancer types**	**Biological effect**	**Mechanism**	**Ref**
Exosomal miR-940	Epithelial ovarian cancer	Suppress anti-tumor immune responses	Promotes M2 polarization of tumor-associated macrophages	(44)
Exosomal miR24-3p	Nasopharyngeal carcinoma	Suppress anti-tumor immune responses	Inhibit T-cell proliferation and differentiation, and the induction of Tregs	(45)
Exosomal TGF-β1	Hypoxic cancer	Suppress anti-tumor immune responses	TGF-β1 downregulates NKG2D and	(46)
and miR23a			miR23a directly targets CD107a	
Exosomal miRLet-7a	Melanoma	Suppress anti-tumor immune responses	Enhanced the oxidative phosphorylation in bone marrow-derived macrophages	(42)
Exosomal miR-10a and miR-21	Glioma	Suppress anti-tumor immune responses	Enhanced expansion and activation of myeloid-derived suppressor cells	(47)
Exosomal TGF-β	Breast cancer	Suppress anti-tumor immune responses	Inhibit T cell proliferation *via* TGF-β, IL-10 and PGE2	(48)
Exosomal HSP70	Oral squamous cell carcinoma	Suppress anti-tumor immune responses	Inhibit T cells through a miR-21/PTEN/PD-L1 regulation axis	(49)

### Macrophage

Tumor-associated macrophages (TAMs) can differentiate into either M1-type macrophages with pro-inflammatory effects or M2-type macrophages with anti-inflammatory effects, both of which are regulated by the TME (50). Furthermore, studies have shown that this polarization of macrophages can be affected by exosomes within the hypoxia TME. Specifically, exosomes derived from different parental cells will promote the polarization of macrophages into different subtypes. For example, the exosomes secreted by renal tubular epithelial cells under hypoxia can promote the polarization of M1 macrophages and induce high levels of inflammatory responses (51). On the other hand, hypoxia can alter miRNAs levels in tumor cell-derived exosomes and leading to M2 polarization of macrophages (44). Similarly, Chen et al. found that hypoxia can stimulate M2 phenotype polarization by upregulating miR-940 expression in epithelial ovarian cancer (EOC)-derived exosomes, while the M2 subtype macrophages can in turn promote the proliferation and migration of EOC cells. In addition, hypoxia can also upregulate the expression of miR-21-3p, miR-125b-5p, and miR-181d-5p in exosomes *via* HIF to induce the M2-polarization of macrophages (24). In another study, Qian et al. found that compared with normoxic glioma cell exosomes, hypoxia glioma cell exosomes had a stronger ability to induce macrophage polarization to the M2 type. Meanwhile, the study also indicated that the miR-1246 level was significantly enriched in the exosomes of hypoxia glioma cells, which could activated the STAT3 pathway and inhibit the NF-κB signaling pathway, thereby promoting the polarization of M2 phenotype (52). Nevertheless, although exosomes secreted by tumor cells under hypoxia stress mostly induce M2 polarization of macrophages, strategies to convert TAMs to a predominantly M1 phenotype have been proposed for novel immunotherapy.

### MDSC

Myeloid-derived suppressor cells (MDSCs) are highly heterogeneous immature cells derived from bone marrow that can inhibit the activation of T cells, promote M2 polarization of macrophages, and inhibit NK cytotoxicity (53). Numerous studies have demonstrated that the activation, expansion, and immunosuppression of MDSCs can be promoted by exosomes. However, whether exosomes under hypoxia conditions would have similar effects on the proliferation and immunosuppression of MDSCs remained to be elucidated. In this regard, researchers found that hypoxia can directly stimulate the expressions of HSP72 and toll-like receptor 2 (TLR2) in exosomes, which can directly participate in the regulation of MDSCs (54, 55). Specifically, Chalmin et al. found that HSP72 in exosomes can mediate the interaction between tumor cells and MDSCs by triggering STAT3 activation (56). Similarly, Xiang et al. found that exosomes released from cultured B16 tumor cells could induce the activation and expansion of MDSC in a TLR2-dependent manner (57). All these results suggest that hypoxia-induced exosomes play a significant role in suppressing tumor immune surveillance by promoting the suppressive function of MDSC.

Although an immunosuppressive milieu mediated by MDSCs has been demonstrated in patients with glioma, the mechanisms of MDSC development and activation have not been elucidated. Guo et al. found that the expression of miR-10a and miR-21 was increased in glioma cells-derived exosomes under hypoxia conditions compared with normoxia (47). Furthermore, they also found that hypoxia-induced glioma cells can stimulate the differentiation of functional MDSCs by transferring exosomal miR-29a and miR-92a to MDSCs (58). Accumulating evidence supports that hypoxia can induce changes in miRNA expression in tumor-derived exosomes, thereby activating MDSCs and enhancing their function to promote tumor growth. This suggests that blocking the immunosuppressive effect of MDSCs may be an effective way to improve the efficacy of immunotherapy. Therefore, clarifying the exact effect of hypoxia on the immunosuppressive function of MDSCs may provide new insights for targeting exosomal secretion and its contents (especially miRNAs and proteins) for anti-tumor immunotherapy.

### T Cell

T cells are considered to be the major cell subset in anti-tumor immunity. Treg cells are a specialized population of T cells thought to suppress anti-tumor immune responses (59). Studies have reported that miR-214 expression was significantly increased in tumor cell-derived exosomes under hypoxia TME (60). Yin et al. observed that exosomes from lung cancer cell lines can effectively transport miR-214 to CD4^+^ T cells, thereby promoting the expansion of Treg cell subsets and the secretion of IL-10 (61). A study by Mrizak et al. found that exosomes released from nasopharyngeal carcinoma cells under hypoxia conditions express the chemokine CCL20, leading to preferential recruitment of Treg cells to tumor sites. In addition, these hypoxia-induced exosomes were also able to induce Treg cell expansion and enhance their immunosuppressive effects (62). Collectively, these results suggest that hypoxia participates in Treg cell-mediated immunosuppression by modulating the cargo of exosomes.

On the other hand, hypoxia-induced exosomes can also promote tumor immune escape by directly inhibiting the activity of T cells. Rong et al. found that TGF-β can be delivered to T cells through breast cancer cell-derived exosomes, thereby inhibiting T cell proliferation, while anti-TGF-β treatment reversed the immunosuppressive effects of the exosomes (48). In addition, HIF can also exert an immunosuppressive effect by upregulating the PD-L1 expression in exosomes. The study by Poggio et al. showed that tumor cells can release PD-L1-carrying exosomes under hypoxia conditions, which can function as an immune checkpoint by binding to PD-1 on the surface of activated T cells (63). In patients with gastric cancer, exosome-carried PD-L1 resulted in decreased CD4^+^ and CD8^+^ T cell infiltration and activity (64). Similarly, higher levels of PD-L1 were found in exosomes from patients with active disease and poor survival (65). Together, these studies suggest the inhibitory effect of hypoxia-induced exosomes on T cell-mediated anti-tumor immunity.

### NK Cell

There is ample evidence that hypoxia-induced exosomes can evade immune surveillance by binding to NK cells. According to literature reports, the activation of NK cells in anti-tumor immunity is mediated by surface-active receptors, such as NKG2D and NKp44 (66). However, hypoxia-induced exosomes reportedly suppress host NK cell cytotoxicity by reducing NKG2D expression, thereby disrupting the host immune system and promoting the formation of a tumor-promoting microenvironment (67). For example, hypoxia TME can increase the levels of TGF-β1, miRNA-210, and miRNA-23a in exosomes secreted by tumor cells. Specifically, the uptake of hypoxia exosomes by NK cells can transfer TGF-β1 in exosomes to NK cells and inhibit the expression of its surface-activated receptor NKG2D, thereby inhibiting NK cell function. Subsequent miRNA analysis showed highly expressed miR-23a in hypoxia-induced exosomes, which can target the expression of CD107a in NK cells, thereby inhibiting the activation of NK cells (46). Similarly, Xia et al. also described that clear cell renal cancer triggers NK cell dysfunction in an exosome-dependent manner, and this inhibitory function also activates the TGF-β/SMAD signaling pathway through TGF-β1 (68). In addition, the researchers found that exosomes isolated from the plasma of patients with melanoma contained a large amount of PD-L1, FasL, and TGF-β. These hypoxia-associated exosomes not only inhibited the activity of CD8^+^ T cells, but also down-regulated *NKG2D* expression in NK cells. In contrast, targeting these exosomes with monoclonal antibodies or pharmacological inhibitors can restore immune cell function (69).

## Exosome-Based Anti-Tumor Immunotherapy

Tumor immunotherapy is a new anti-cancer strategy that kills tumor cells by activating immune cells or restoring exhausted immune cells. As an important regulator of the TME, exosomes-based therapies are considered a promising approach to promoting tumor immunotherapy. For example, exosomes secreted by tumor cells contain a vast number of tumor-associated antigens, such as MHC I and MHC II, that can be used as a tumor vaccine to promote anti-tumor immune responses (70). Hsu et al. found that dendritic cell (DC)-derived exosomes can be loaded with a variety of polypeptide antigens (such as MHC I, MHC II) and co-stimulatory molecules that contribute to the initiation and activation of T cells, such as CD80, CD83, CD86, etc. (71). In a mouse model of pancreatic cancer, subcutaneous injection of exosome-loaded DC vaccine extended survival in mice, while combination therapy with chemotherapeutics such as of gemcitabine simultaneously reduced tumor MDSCs content and increased the activation of T cells (72). In addition, the use of DC cell-derived ovalbumin exosomes to stimulate CD4^+^ T cells can inhibit the differentiation and proliferation of Treg cells and promote the formation of memory CD8^+^ T cells. This represents another attractive exosome-based anti-tumor vaccine option (73). In addition, DC-derived exosomes contain NKG2D ligands. Therefore, Viaud et al. reported that DC vaccines can activate NK cells and release TNF (40). However, due to the cargo complexity of exosomes, exosome-based tumor vaccine strategies may also inhibit anti-tumor immunotherapy by inducing apoptosis of activated CD8^+^ T cells (74). For example, exosomes can impair the activation of T cells by IL-2 and promote the proliferation of Treg cells for immunosuppressive effects (75). Nevertheless, it is still possible to consider optimizing the use of exosome vaccines by modifying the exosomes and adjusting their dosage. For example, Samuel et al. used IFN-γ to stimulate the secretion of exosomes from mature dendritic cells which increased the expression of co-stimulatory molecules in exosomes to enhance T cell activation (74).

## Conclusion

In recent years, anti-tumor immunotherapy represented by immune checkpoint inhibitors has changed the treatment option for various tumor types. As mentioned earlier, the infiltration and activation of immune cells in the TME are closely related to successful immunotherapy. Therefore, understanding the impact of exosomes on the anti-tumor immune system can further enhance the effect of immunotherapy. In fact, hypoxia-induced exosomal features have been extensively studied and consensus has been reached. First, hypoxia-induced exosomes are not vesicles loaded with cellular waste, but key mediators of intercellular communication. Second, in the adverse TME such as hypoxia, high glucose and drug therapy, the cargo carried and delivered in exosomes is significantly altered, which in turn modulates immune cell function (76, 77). Third, exosomes secreted by immune cells such as DCs and chimeric antigen receptor T cells (CAR T cells) can inhibit the growth, proliferation, and metastasis of tumor cells (78). Therefore, the preparation of exosome-based tumor vaccines to enhance tumor antigen presentation and modulate immune responses in the TME is a potential avenue for therapeutic development.

## Further Perspective

While initial preclinical studies have shown promising results, clinical trials have failed to achieve comparable results. This suggests that there are still unresolved challenges with existing exosome treatments (79). For example, existing exosome therapeutics have low targeting efficiency and are easily engulfed by the immune system. In addition, the current exosome isolation methods are expensive, and large-scale exosome isolation technology still needs to be developed (80). Therefore, these problems would need to be solved before clinical application. Moreover, exosome-based immunotherapy is currently still in early clinical trials, and there are no specific international guidelines for the production and application of this novel therapeutic. Future studies should investigate the effects of hypoxia on the formation, release, and cargo components of exosomes, as well as the mechanism by which the hypoxia TME mediates the regulation of immune cell function by exosomes. These studies would not only provide insight into the poor response of cancer immunotherapy regimens in current clinical trials, but may also serve as a reference for exosome application in anti-cancer drug delivery to improve anti-tumor therapy precision.

## Author Contributions

WG and TQ drafted the manuscript and carried out the figures in manuscript.literature research of the review, figures and drafted the manuscript. BD performed literature analysis of the review and editing the final manuscript. TL, QL, and XX provided supervision and contributed to the conceptualization of the review. All authors read and approved the published version of the manuscript.

## Conflict of Interest

The authors declare that the research was conducted in the absence of any commercial or financial relationships that could be construed as a potential conflict of interest.

## Publisher’s Note

All claims expressed in this article are solely those of the authors and do not necessarily represent those of their affiliated organizations, or those of the publisher, the editors and the reviewers. Any product that may be evaluated in this article, or claim that may be made by its manufacturer, is not guaranteed or endorsed by the publisher.
